# Atypical clinical presentation of Ebola virus disease in pregnancy: Implications for clinical and public health management

**DOI:** 10.1016/j.ijid.2020.05.064

**Published:** 2020-08

**Authors:** Boris I. Pavlin, Andrew Hall, Jan Hajek, Muhammad Ali Raja, Vikas Sharma, Otim Patrick Ramadan, Sharmistha Mishra, Audrey Rangel, Aileen Kitching, Katrina Roper, Tim O’Dempsey, Judith Starkulla, Amy Elizabeth Parry, Rashida Kamara, Alie H. Wurie

**Affiliations:** a World Health Organization, PO BOX 5896, Port Moresby, NCD 111, Papua New Guinea; b King's College London, Strand, London WC2R 2LS, United Kingdom; c University of British Columbia, Vancouver, BC V6T 1Z4, Canada; d World Health Organization, PO BOX 529, Freetown, Sierra Leone; e World Health Organization, 537, A Wing, Nirman Bhawan, Maulana Azad Road, New Delhi 110011, India; f World Health Organization, Juba, South Sudan; g St. Michael's Hospital, 30 Bond St, Toronto, ON M5B 1W8, Canada; h International Medical Corps, Lunsar, Port Loko, Sierra Leone; i Public Health England, Wellington House, 133-155 Waterloo Rd, Lambeth, London SE1 8UG, United Kingdom; j World Health Organization, Rue Jean Baldassini, 69007 Lyon, France; k Liverpool School of Tropical Medicine, Pembroke Pl, Liverpool L3 5QA, United Kingdom; l World Health Organization, #61-64, Street, 306 Corner Preah Norodom Blvd (41), Phnom Penh, Cambodia; m Ministry of Health and Sanitation, Youyi Building, Freetown, Sierra Leone

**Keywords:** Ebola, Pregnancy, Sierra Leone, Outbreak, Epidemic

## Abstract

**Background:**

Between December 2013 and June 2016, West Africa experienced the largest Ebola virus disease (EVD) outbreak in history. Understanding EVD in pregnancy is important for EVD clinical screening and infection prevention and control.

**Methods:**

We conducted a review of medical records and EVD investigation reports from three districts in Sierra Leone. We report the clinical presentations and maternal and fetal outcomes of six pregnant women with atypical EVD, and subsequent transmission events from perinatal care.

**Results:**

The six women (ages 18–38) were all in the third trimester. Each presented with signs and symptoms initially attributed to pregnancy. None met EVD case definition; only one was known at presentation to be a contact of an EVD case. Five women died, and all six fetuses/neonates died. These cases resulted in at least 35 additional EVD cases.

**Conclusions:**

These cases add to the sparse literature focusing on pregnant women with EVD, highlighting challenges and implications for outbreak control. Infected newborns may also present atypically and may shed virus while apparently asymptomatic. Pregnant women identified a priori as contacts of EVD cases require special attention and planning for obstetrical care.

## Highlights

• Pregnant women infected with Ebola virus may present atypically.
• Current Ebola virus disease case definitions are inappropriate for pregnant women.
• Infected newborns may appear asymptomatic despite being potentially infectious.
• Obstetric care during an Ebola outbreak requires heightened precautions.
• Pregnant contacts of Ebola-infected individuals require special considerations.

## Background

Between December 2013 and June 2016, West Africa experienced the largest outbreak of Ebola virus disease (EVD) in history, with at least 28,646 cases and 11,323 deaths (WHO, 2019). Prior to this outbreak, the small number (2,345) of cumulative cases of EVD meant that relatively little was known about the clinical course of EVD (CDC, 2019), especially in pregnant women. Few reports describe the clinical outcomes in pregnant women infected with EVD, and their newborns (Piot et al., 1978, World Health and Organization, 1978, Bwaka et al., 1999, Mupapa et al., 1999, Caluwaerts et al., 2016, Caluwaerts et al., 2018, Baize et al., 2014, Caluwaerts, 2017, Chiu et al., 2018, Henwood et al., 2017, Lyman et al., 2018, Mpofu et al., 2019, Nelson et al., 2016, Oduyebo et al., 2015, UNFPA, 2020). Previous reports largely described pregnant women with EVD who presented with typical symptoms, spontaneous abortion (World Health and Organization, 1978, Bwaka et al., 1999, Mupapa et al., 1999, Baize et al., 2014, Henwood et al., 2017, Lyman et al., 2018, Mpofu et al., 2019, Baggi et al., 2014, Chertow et al., 2014, Schieffelin et al., 2014), pregnancy-related haemorrhage (Mupapa et al., 1999, Caluwaerts et al., 2016, Caluwaerts, 2017, Henwood et al., 2017, Lyman et al., 2018, Mpofu et al., 2019, Schieffelin et al., 2014), and stillbirth (Mupapa et al., 1999, Caluwaerts et al., 2016, Henwood et al., 2017, Lyman et al., 2018, Mpofu et al., 2019, Oduyebo et al., 2015, Baggi et al., 2014). While mortality has historically been very high among pregnant women with EVD (averaging 86% in a review by Bebell et al. of 111 patients) (Bebell et al., 2017), there are increasing reports of maternal survival, usually but not universally accompanied by fetal loss (World Health and Organization, 1978, Bwaka et al., 1999, Mupapa et al., 1999, Caluwaerts et al., 2016, Caluwaerts et al., 2018, Baize et al., 2014, Caluwaerts, 2017, Chiu et al., 2018, Henwood et al., 2017, Lyman et al., 2018, Mpofu et al., 2019, Oduyebo et al., 2015, UNFPA, 2020). We are aware of four previous reports each involving a single pregnant woman with EVD who presented in labour without fever or typical symptoms of EVD (Akerlund et al., 2015, Okoror et al., 2018, Bower et al., 2016, Dunn et al., 2016).

Understanding EVD in pregnancy is important for clinical EVD screening. Symptoms associated with pregnancy and labour can mimic EVD (Deaver and Cohen, 2015). EVD in pregnancy is also important for infection prevention and control (IPC) because in addition to transplacental EVD transmission, fetal and horizontal transmission (for example, to birth attendants) can occur via contact with Ebola virus (EBOV) in the products of conception, such as amniotic fluid and the placenta (Mupapa et al., 1999, Oduyebo et al., 2015, Baggi et al., 2014, Muehlenbachs et al., 2017). EBOV testing from some cases suggests that the amniotic fluid remains a reservoir for Ebola virus persistence even after maternal symptoms resolve and the virus is no longer detectable in peripheral blood (Baggi et al., 2014, Muehlenbachs et al., 2017).

In May 2015, several districts in Sierra Leone began to systematically collect data on pregnancy among all patients with EVD. We observed atypical clinical presentations of EVD among several of the pregnant women. Here we report the clinical presentations and the maternal and fetal outcomes of six pregnant women in Sierra Leone who did not meet the World Health Organization (WHO) EVD case definition in use at the time,^1^ and the secondary transmission events from obstetric and/or neonatal care. We discuss the urgent implications of these findings on clinical and public health practice in EVD outbreaks.

## Methods

We reviewed all available medical records from health facilities that treated patients with EVD in three of the most EVD-affected districts of Sierra Leone: Makeni General Hospital in Bombali district; Maforki Ebola Treatment Centre (ETC) and the International Medical Corps ETC in Port Loko district; and Princess Christian Maternity Hospital and Aberdeen Women's Centre, in Freetown. We also reviewed all EVD case investigation reports from the same districts. We selected all records for women with EVD who were reported as pregnant and who did not meet the EVD case definition. Six such records were identified, and their relevant clinical and epidemiologic characteristics were summarized in a case series (Table 1).

**Table 1:** Clinical course and epidemiologic features of pregnant women with atypical Ebola virus disease

Case	District of residence	Month/year	At presentation				Clinical outcomes						EBOV exposure history	Public health outcomes (2° cases)
			Age	Stage of pregnancy	Symptoms & signs	Met EVD case defn.	Fetal/pregnancy outcome	Reason for suspicion of EVD	Maternal outcome	Maternal EBOV test result	Neonatal outcome	Neonate EBOV test result		
	Bombali	November 2014	25 yrs	T3	Labor pains	No	Live birth by SVD in MGH	PPH at 1 day post-delivery (still afebrile)	Died at 1 day post-delivery	p-m oral swab EBOV (+)	discharged to family; died	Not done	Hx of QH, gave false address at presentataion.	1 other patient infected by the mother; unknown number of family members infected by the newborn
	Port Loko	December 2014	18 yrs	T3	Labor pains, V, D, H, muscle pain; afebrile initially but T° during labor	No (suspected by clinical judgement)	Live birth by SVD at PCMH EVD Isolation Unit	Based on symptoms	PPH post-delivery, died	Blood EBOV (+)	Became symptomatic at day 15, died day 19	Blood EBOV (+)	Unknown	None
	Kambia	January 2015	35 yrs	T3	Labor pains	No	Live birth by SVD at PCMH	Nil at time of delivery	Discharged home post-delivery; became febrile unknown days later; died in RTA	Blood EBOV −ve × 2 at time of delivery; p-m oral swab EBOV (+)	Became unwell at unknown day of life, died next day	p-m oral swab EBOV (+)	In QH at time of delivery	23 (1 family member, resulting in 17 confirmed & 5 suspect cases)
	Port Loko	February 2015	38 yrs	T3	Labor pains	No	Live birth by SVD at home	PPH few hrs post-delivery	Died on day of delivery	p-m oral swab EBOV (+)	Became unwell at day 7, died day 8	EBOV −ve at 3 days old, (+) at 5 days old	Visited QH of family member	9 household contacts (5 died; 4 survived)
	Freetown	May 2015	19 yrs	T3	Fetal death	No	Induced delivery of stillborn in hospital	None at time of delivery	Discharged home asymptomatic	EBOV −ve from blood sample	Stillborn	EBOV (+) from oral swab	None identified	None identified
	Port Loko	May 2015	20 yrs	T3	Labor pains	No	Live birth by SVD at home	None at time of delivery	Died on day of delivery	p-m oral swab EBOV (+)	Became unwell at day 7, died day 11	EBOV (+) from oral swab	Patient's mother had EVD	4 (2 caregivers, 2 children)

AP = abdominal pain; D = diarrhoea; EVD = Ebola virus disease; EBOV = Ebola virus; HCW: healthcare worker; H = headache; MGH = Makeni General Hospital (Bombali); p-m = post-mortem; PCMH = Princess Christian Maternity Hospital (Freetown); PHU = peripheral health unit; PPH = post-partum haemorrhage; PV = per vagina; QH = quarantined home; RTA = road traffic accident; SVD = spontaneous vaginal delivery; T° = temperature; T = trimester of pregnancy; V = vomiting; (+) = positive.

The proportion of pregnant women who did not meet the EVD case definition was not calculated because the total number of EVD-affected pregnant women (i.e. the denominator) was unavailable due to inconsistent reporting of pregnancy status, particularly in records prior to May 2015.

## Ethical approval

Approval for this study was obtained from the Sierra Leone Ethics and Scientific Review Committee. As the study involved retrospective review of records, no individual patient consent was obtained.

## Case series

### Case 1

#### Initial presentation and clinical course

In November 2014, a 25-year-old female in the third trimester of pregnancy presented to Makeni General Hospital in Bombali district with abdominal pain consistent with labour. She had no other symptoms, was afebrile, had normal conjunctivae, and appeared well. At the time of admission, systematic EVD screening was not in place at the facility and she was not questioned about potential EVD exposures. Two hours after admission, the neonate was delivered by uncomplicated spontaneous vaginal delivery. The neonate appeared healthy and was feeding well. The nurse who performed the delivery wore routine obstetrical personal protective equipment (PPE) including a disposable gown and gloves.

Unexpectedly, 12 hours later, the patient developed profuse vaginal bleeding and required a blood transfusion. Despite administration of ergometrine and oxytocin, the vaginal bleeding persisted and she began bleeding at the site of the peripheral intravenous cannula. Although she remained afebrile, the bleeding alerted medical staff to the possibility of EVD. She was moved to an EVD isolation ward attached to the hospital pending transfer to a facility for patients suspected of EVD. However, she continued to bleed and died of post-partum haemorrhage several hours later. As per standard practice for all deaths during this time, post-mortem reverse transcriptase polymerase chain reaction (RT-PCR) testing was performed on an oral swab and found to be positive for EBOV.^2^ The neonate was never tested for EBOV and was discharged home to the care of family members in the father's village, as there was no suspicion of EVD. The products of conception (amniotic fluid, placenta) were not tested for EBOV.

#### EBOV exposure

Subsequent epidemiologic investigation uncovered that the patient was from a quarantined village and had been identified as a possible contact of a known case of EVD within the 21 days prior to admission. When labour began, she broke her quarantine, travelled to the hospital, and used her husband's address from a different village because of fear that she would otherwise be denied health care.

#### Secondary cases

None of the healthcare staff involved in the delivery developed EVD. However, contact investigations revealed that another pregnant woman who delivered a neonate at the same time and in the same delivery room, and was cared for by the same health-care providers, died of EVD twelve days post-partum and was likely infected through nosocomial transmission.

The baby became ill and died soon after discharge. Direct child-care and a traditional burial of the baby triggered an outbreak of an unknown number of additional EVD cases in the case patient's husband's village.

### Case 2

#### Initial presentation and clinical course

In December 2014, an 18-year-old pregnant female presented to the Maforki Ebola Treatment Centre (ETC) in Port Loko district, almost at full term and in the initial stages of labour. She was afebrile, but was experiencing abdominal pain, vomiting, muscle pain, headache and diarrhoea. She denied a history of known EVD exposure; thus, lacking either fever or EVD exposure, she did not meet the case definition despite having multiple other symptoms of EVD. She was transferred to the Princess Christian Maternity Hospital (PCMH)’s EVD isolation unit in Freetown for higher-level obstetrical care and because of a clinical suspicion of EVD despite not meeting the case definition. EBOV RT-PCR testing on admission was positive. At the hospital, she became febrile (39 °C) and delivered via spontaneous vaginal delivery, assisted by health-care providers in full PPE. She developed severe post-partum haemorrhage and hypovolemic shock, and died on the day of admission. The products of conception were not tested for EVD.

The healthy newborn was transferred to the Port Loko Observational Interim Care Centre (an observation Centre for asymptomatic children of EVD-infected parents or guardians). On day 15 of life, the neonate became unwell and was isolated to the suspect ward of the Maforki Ebola Treatment Centre (ETC). Blood tests were positive for EBOV by RT-PCR. The neonate died on day 19.

#### EBOV exposure

The source of the mother's infection could not be determined.

#### Secondary cases

None detected.

### Case 3

#### Initial presentation and clinical course

In January 2015, a 35-year-old female in her third trimester of pregnancy went into labour while quarantined at her home in Kambia district as a contact of an EVD case. The patient was brought by ambulance to a local Holding Centre (a facility used for holding suspect cases pending their laboratory result), out of precaution because she was a contact, not out of suspicion of EVD. She tested negative for EBOV. She was transferred to PCMH's isolation unit in Freetown, where she again tested negative and subsequently delivered a healthy neonate. The delivery was assisted by health-care providers in full PPE. The patient was discharged and immediately returned to Kambia to a neighbouring village, as her home was still under quarantine. The products of conception were not tested for EVD.

Within a few days of discharge, she developed a fever. On route to hospital, her motorbike was involved an accident and she died at the scene. A post-mortem oral swab was positive for EBOV. Two days later, the neonate fell ill and with conjunctivitis and fever. The neonate was returned to Princess Christian Maternity Hospital in Freetown and died shortly after admission. A post-mortem swab of the neonate was EBOV-positive.

#### EBOV exposure

Patient was a known contact in a quarantined home.

#### Secondary cases

Investigation revealed that no attending healthcare developed EVD. However, a family member who may have cared for the patient and the neonate post-partum was infected. The family member's infection triggered a transmission chain of 23 onward cases.

### Case 4

#### Initial presentation and clinical course

In February 2015, a 38-year-old female in Port Loko district delivered a healthy neonate at home at 36 weeks gestation. She reported no symptoms other than labour pains prior to delivery; this was later corroborated by family members. The delivery was complicated by post-partum haemorrhage the following day, and the patient died approximately four hours later. The delivery and post-partum care was assisted by two women at home. No gloves or other PPE were used during the delivery, post-partum care, discarding of tissues, washing of the neonate, or cleaning of the delivery area (linens). The remaining products of conception were reportedly discarded in the latrine, including the placenta, which was not discoloured and appeared healthy. A post-mortem oral swab was positive for EBOV, and reported to the Ebola surveillance team when the newborn was 3 days old.

The newborn was described as feeding well and showing no symptoms of EVD. However, the Ebola response team felt that the newborn was likely to have been infected during gestation or childbirth. Thus, the newborn was taken to the International Medical Corps ETC, and into a single ward (with no other patients), as a precautionary measure to enable early EVD detection/diagnosis and interrupt onward transmission. This ETC was chosen because of the availability of nearly 24-hour care by EVD survivors wearing light PPE (disposable gowns and gloves). The EVD survivor care enabled near-constant hands-on supportive and nutritional care, in addition to the clinical care provided by the other staff in full PPE.

Admission blood tests, on day 4 of life, were RT-PCR-negative for EBOV. A repeat blood RT-PCR on day 6 of life was positive. Throughout this time, the newborn still appeared well, was feeding fell with wet diapers, and had no detectable signs of EVD. At seven days of age, the neonate suddenly became unwell, developed a fever, and died on the following day.

#### EBOV exposure

Subsequent epidemiologic investigation found that 10–12 days prior to delivery, the pregnant patient had been visiting the quarantined home of her step-mother while the step-mother was symptomatic with EVD. Thus, the pregnant patient was a contact of a known EVD case, but the exposure was only discovered after the post-mortem EVD diagnosis.

#### Secondary cases

One woman who assisted in the birth, as well as her son, developed EVD; the woman died. The case patient's sister and her husband also developed EVD and died, as did another female relative who lived with the case patient. Another man, and a woman and her two sons, who lived in the house where the baby was born, were infected; the man died. This brought a total of five deaths and four survivors as secondary cases.

### Case 5

#### Initial presentation and clinical course

In May 2015 a 19-year-old female from Freetown presented at the Aberdeen Women's Centre at 36 weeks gestation after noting an absence of fetal movement for several days. An ultrasound documented fetal demise. The patient underwent induction of labour, and two skilled birth attendants in PPE assisted with the delivery.

The mother was discharged home the day of delivery. As per routine procedure for all deaths, an oral swab was taken of the neonate. The swab tested positive for EBOV. Coincidentally, on the following day, the mother returned to the Centre to collect prescribed medications; she was tested for EBOV by RT-PCR and found to be negative. Further antibody testing of the mother indicated presence of IgG and negative for IgM, confirming that she was an EVD survivor with subclinical illness at some point prior to delivery.

#### EBOV exposure

The source of the mother's infection could not be determined.

#### Secondary cases

None detected.

### Case 6

In May 2015, a 20-year old female in Port Loko district delivered a healthy newborn at 34 weeks of gestation. The family and birth attendants reported that prior to delivery, the patient was experiencing episodic abdominal pain consistent with labour, without any other symptoms.

Four women assisted with the home-delivery, including a traditional birth attendant. All reported wearing gloves while providing care and post-delivery cleaning. Approximately an hour after delivery, the patient died unattended. There had been no post-partum haemorrhage, no observed maternal seizure activity or change in mental status. The placenta was not discoloured. It was discarded in a latrine. The family reported the death as an alert, and the burial team conducted a post-mortem Ebola virus oral swab for RT-PCR and safe burial of the patient. The result came back positive.

The neonate became unwell at home on day 7 of life, and died on day 11. The neonate was not taken to an ETC for testing and care, because the household and community refused transfer. The neonate received a safe burial, and was diagnosed with EVD by post-mortem swab.

#### EBOV exposure

During the case investigation, it was uncovered that the patient had been in contact with her mother who had recently died of EVD.

#### Secondary cases

One caregiver of the neonate and two of the caregiver's children who resided in the same quarantine home were infected with EBOV from exposure to the neonate. An additional caregiver who had exposure to both the mother and the neonate also became infected.

## Discussion

Pregnant women require special attention during an EVD outbreak. As reported in this case series, the clinical presentation of EVD in pregnant women may be atypical and difficult to recognize, compromising appropriate care and posing a transmission risk to caregivers. Providing care for the neonates poses additional risks. In all instances, the neonates were incubating Ebola virus, and at least two were documented to be asymptomatic while viremic, and thus – infectious while asymptomatic. EVD in neonates was universally fatal in this series. Our findings are particularly timely in light of the large ongoing EVD outbreak in Democratic Republic of the Congo, in at least 115 pregnant women with probable or confirmed EVD have been identified (EVD in DRC response team, preliminary data). Our findings raise three important issues about the clinical and public health management of pregnant women during an EVD outbreak.

### Case definition

As these cases illustrate, pregnant women who subsequently test positive for EBOV may initially present in labour without fever or typical symptoms of EVD; hence, current EVD case definitions lack sensitivity for pregnant women. The apparent lack of typical EVD symptoms may be because the immunologic changes associated with pregnancy alter the manifestations of EVD; because the normal symptoms of pregnancy, like abdominal pain, may overshadow and mask those of EVD; because pregnant women may be reluctant to admit they have symptoms of EVD; or a combination of these factors. Importantly, only one case patient was identified at the time of presentation as having an epidemiologic link to a known case and was under surveillance; others might have concealed their history or had no known history of EVD exposure. This illustrates that the epidemiologic criterion for suspicion of EVD is unreliable as a means of screening, and that heightened IPC measures should be applied to all pregnant women presenting for obstetrical care in an Ebola outbreak.

Infected newborns may also not manifest typical signs of EVD, and may even remain asymptomatic despite having positive blood tests for EBOV by RT-PCR (and therefore being potentially infectious). These findings are consistent with the initial reports by Piot et al. of EVD in 10 infants born to women who died of EVDin which the infants died without recognition of a preceding illness typical of EVD (Piot et al., 1978) (recognizing that laboratory confirmation of EVD was not available in these infants).

In the West Africa outbreak, as in previous ones, questions about pregnancy had not been routinely incorporated into epidemiologic case investigation forms; this remains the case for the standard epidemiologic case investigation form in use in the current DRC outbreak. Given the impact of pregnancy on EVD, we strongly recommend that specific pregnancy-related questions be incorporated into EVD case investigation forms.

### Obstetric care

In Sierra Leone, varying approaches were undertaken to mitigate the potential risk posed by pregnant women with EVD: staff at some facilities wore full PPE for all deliveries, regardless of EVD case or contact status; in a limited number of other facilities (e.g. PCMH), staff performed blood tests for EBOV RT-PCR on all pregnant women who arrived in labour from select high incidence districts. This latter approach was only feasible because of the on-site presence of rapid EBOV testing capability, and sufficient laboratory capacity to absorb the higher workload. It should be noted that staff followed contact precautions with gowns and gloves regardless of testing results. Garde, et al. have recently published a detailed description of the procedures undertaken at PCMH (Garde et al., 2019).

All health facilities which provide obstetrical care should be prepared, and receive necessary training, to safely care for pregnant women during an EVD outbreak using appropriate IPC precautions. Given the limited sensitivity of existing case definitions and laboratory tests, strict contact precautions should always be followed. Women who are identified as contacts and those who meet the EVD suspect case definition should ideally be cared for and delivered in ETCs. If circumstances permit, during an EVD outbreak, all deliveries should have RT-PCR testing (onsite or offsite) of maternal blood, amniotic fluid and umbilical cord blood. Babies born to mothers with EVD are at very high risk and should be cared for with contact precautions and closely monitored for 21 days. Our recommendations are consistent with recently issued WHO guidelines for the management of pregnant and breastfeeding women in the context of Ebola virus disease (WHO, 2020).

### Pregnant contacts

From March 2015, in Port Loko district, all willing asymptomatic pregnant women from quarantined homes were admitted for observation at maternity Holding Centres such as PCMH for the duration of their 21-day monitoring period as EVD contacts. This approach was based on several tenets: (1) that appropriate antenatal care (ANC) was not possible while in quarantine; (2) that a pregnant contact who miscarried or went into labour while in a quarantined home would have difficulty obtaining timely and safe medical care and could expose household members to large amounts of potentially infectious bodily fluids; and (3) routine health services often refused to care for pregnant women in labour or with vaginal bleeding during the Ebola outbreak.

This precautionary approach was not without challenges, including convincing asymptomatic women to be admitted and relatively isolated from others in an EVD facility. While maternity waiting homes next to district hospitals previously existed as an accepted strategy for ensuring timely access to clinic-based obstetrical case in rural areas of Sierra Leone, the widespread quarantines imposed during the West Africa outbreak had a decidedly negative impact on this approach. A Médecins Sans Frontières survey of eight pregnant women found that “women do not want to come to an ETC for [antenatal care] and delivery” [unpublished data]. Admitting asymptomatic and potentially uninfected women into an EVD isolation facility along with confirmed EVD cases could result in nosocomial spread, with risks to the individual as well as public trust. These risks could be mitigated by creating specialized maternity care sections in ETCs designated for antenatal care of asymptomatic pregnant women.

In late April of 2015, national policy was developed to harmonize the approaches discussed above. An agreement was reached to enumerate all pregnant women identified as contacts of EVD cases. Rather than transporting these pregnant women to other facilities for antenatal care, it was decided that they would remain under quarantine and mobile medical teams would provide antenatal care through individual home visits. For example, in Port Loko, a Mother and Baby Unit was established by the non-governmental organization GOAL for all pregnant women in quarantined homes. If such women required any care within their 21 days, including uncomplicated deliveries, they were transferred to the unit for treatment.

## Conclusion

The large numbers of cases in the West Africa EVD outbreak provided an unprecedented opportunity for observations of EBOV transmission and the clinical course of EVD illness and recovery. Our cases add to the sparse literature focusing on pregnant women in this context, highlighting several challenges and implications for outbreak control.

## Disclaimer

The authors alone are responsible for the views expressed in this article and they do not necessarily represent the views, decisions or policies of the institutions with which they are affiliated.

## Conflict of interest

The authors declare no conflicts of interest.

The authors alone are responsible for the views expressed in this article and they do not necessarily represent the views, decisions or policies of the institutions with which they are affiliated.

## Funding

None declared.

## Footnotes

1 The official definition of a suspect case was unexplained bleeding or miscarriage; OR, unexplained death; OR, fever (≥38·0^o^ C) and three or more of the symptoms/signs of headache, abdominal pain, generalized or articular pain, difficulty in swallowing, intense fatigue, difficulty in breathing, nausea or vomiting, hiccups, loss of appetite, miscarriage, or diarrhoea; OR, fever and contact with a clinical (suspect, probable or confirmed) EVD case; OR, three or more symptoms and contact with a clinical case (Sierra Leone Ministry of Health Sanitation, 2014). A probable case, while not officially defined, was most commonly either a suspect case that was known to have had contact with a clinical case; OR, a patient that was, on clinical and/or epidemiological grounds, very likely to have EVD.

2 RT-PCR cycling times were not recorded in medical records for any of the six patients described.
